# Admission vital signs as predictors of COVID-19 mortality: a retrospective cross-sectional study

**DOI:** 10.1186/s12873-022-00631-7

**Published:** 2022-04-29

**Authors:** Ahmed Sameer Ikram, Somasundram Pillay

**Affiliations:** 1 King Edward VIII Hospital, Durban, South Africa; 2 Lecturer Nelson R Mandela School of Clinical Medicine, King Edward VIII Hospital, Durban, South Africa

**Keywords:** COVID-19, Vital signs, Oxygen saturation, Respiratory rate, Blood pressure, Heart rate, Glucose, Temperature, Age, Mortality

## Abstract

**Background:**

COVID-19 remains a major healthcare concern. Vital signs are routinely measured on admission and may provide an early, cost-effective indicator of outcome – more so in developing countries where such data is scarce. We sought to describe the association between six routinely measured admission vital signs and COVID-19 mortality, and secondarily to derive potential applications for resource-limited settings.

**Methods:**

Retrospective analysis of consecutive patients admitted to King Edward VIII Hospital, South Africa, with COVID-19 during June–September 2020 was undertaken. The sample was subdivided into survivors and non-survivors and comparisons made in terms of demographics and admission vital signs. Univariate and multivariate analysis of predictor variables identified associations with in-hospital mortality, with the resulting multivariate regression model evaluated for its predictive ability with receiver operating characteristic (ROC) curve analysis.

**Results:**

The 236 participants enrolled comprised 153(77.54%) survivors and 53(22.46%) non-survivors. Most participants were Black African(87.71%) and female(59.75%) with a mean age of 53.08(16.96) years. The non-survivor group demonstrated a significantly lower median/mean for admission oxygen saturation (%) [87(78–95) vs. 96(90–98)] and diastolic BP (mmHg) [70.79(14.66) vs. 76.3(12.07)], and higher median for admission respiratory rate (breaths/minute) [24(20–28) vs. 20(20–23)] and glucose (mmol/l) [10.2(6.95–16.25) vs. 7.4(5.5–9.8)]. Age, oxygen saturation, respiratory rate, glucose and diastolic BP were found to be significantly associated with mortality on univariate analysis. A log rank test revealed significantly lower survival rates in patients with an admission oxygen saturation < 90% compared with ≥90% (*p* = 0.001). Multivariate logistic regression revealed a significant relationship between age and oxygen saturation with in-hospital mortality (OR 1.047; 95% CI 1.016–1.080; *p* = 0.003 and OR 0.922; 95% CI 0.880–0.965; *p* = 0.001 respectively). A ROC curve analysis generated an area under the curve (AUC) of 0.778 (*p* < 0.001) when evaluating the predictive ability of oxygen saturation, respiratory rate, glucose and diastolic BP for in-hospital death. This improved to an AUC of 0.832 (*p* < 0.001) with the inclusion of age.

**Conclusion:**

A multivariate regression model comprising admission oxygen saturation, respiratory rate, glucose and diastolic BP (with/without age) demonstrated promising predictive capacity, and may provide a cost-effective means for early prognostication of patients admitted with COVID-19 in resource-limited settings.

**Supplementary Information:**

The online version contains supplementary material available at 10.1186/s12873-022-00631-7.

## Background

Coronavirus Disease 2019 (COVID-19), caused by the severe acute respiratory syndrome coronavirus 2 (SARS-CoV-2), remains a major healthcare concern globally despite nearly 2 years since inception [1]. Many countries around the world, including South Africa, have experienced recurrent waves of this outbreak, and thus attempts have been made to better delineate factors predicting a poor prognosis. Of particular interest, vital signs of patients admitted with COVID-19 have gained increasing relevance as a potential early marker of outcome.

Despite numerous studies describing the clinical characteristics of patients admitted with SARS-CoV-2 infection, there is a general paucity of data concerning the association between *admission* vital signs and mortality.

Low oxygen saturation and elevated respiratory rate have been consistently associated with severe disease in patients hospitalised with COVID-19 [2–5]. Even on admission, derangements in these vital signs appear to be significantly associated with critical illness and mortality [3–6]. A retrospective analysis of 6180 patients with COVID-19 in the United States revealed significantly higher odds of deaths in patients with a lower oxygen saturation and higher respiratory rate on admission [6]. This is consistent with findings by Zhou et al. demonstrating significantly greater odds of death in patients with a baseline respiratory rate > 24 breaths per minute (adjusted Odds Ratio 10.89; *p* = 0.019) [5].

Similarly, admission hyperglycaemia has been described as a strong predictor of mortality. A metanalysis comprising 16 observational studies and 6386 COVID-19 patients revealed a more than three times greater risk of mortality in patients with admission hyperglycaemia compared with control (95% CI 2.26–5.26) [7]. Interestingly, this association appears to hold even in the absence of diabetes [8, 9].

The evidence for baseline temperature as a predictor of COVID-19 mortality is more conflicting [10, 11]. Most research reveals no significant association between an *elevated* temperature on admission and mortality [10, 11]. Conversely however, Tharakan et al. analysed body temperature in 7614 patients with COVID-19 and identified *hypothermia* as a poor prognostic marker with a mortality rate of 26.5% in patients with an admission temperature < 36 °C, and 44% in those < 35.5 °C [12].

Cardiac involvement and myocarditis represent an important manifestation of SARS-CoV-2 infection as it portends an elevated risk of in-hospital mortality (51% vs. 4%) [13]. Generic sepsis-related risk scores (including Sequential Organ Failure Assessment [SOFA], qSOFA, Systemic Inflammatory Response Syndrome [SIRS] criteria, Modified Early Warning Score [MEWS], National Early Warning Score [NEWS]) [14] have been used in clinical settings to prognosticate and guide management, and feature heart rate and blood pressure abnormalities as key components. Despite this, data on *admission* blood pressure and heart rate associations with COVID-19 mortality is scarce. Even so, several mortality prediction models/scores in COVID-19 utilise heart rate and blood pressure as key components, with a trend towards low systolic blood pressure and elevated heart rate as predictors of poor prognosis [15, 16]. Caillon et al. however, demonstrated an elevation in admission systolic blood pressure as an important component in mortality prediction [17].

There remains a paucity of data from an African perspective. A chart analysis of 92 deceased patients in Ethiopia revealed hypoxia, tachycardia and fever as the most common abnormal vital signs on presentation (60.5, 52.9 and 32% respectively) [18]. Additionally, 17.6 and 20% of patients had hypotension and hypothermia respectively [18]. Another small prospective study of 25 patients hospitalized with COVID-19 in Ghana revealed an association between elevated systolic blood pressures and mortality [19]. However the sample size was small and showed no independent risk factors for mortality after adjustment.

The dearth of data from South Africa is even more prominent. A small observational study in Cape Town comprising 56 patients admitted to the intensive care unit (ICU) demonstrated no significant difference in vital signs (heart rate, systolic and diastolic blood pressure, oxygen saturation and respiratory rate) between survivors and non-survivors [20].

Resource limitations coupled with the emergence of novel strains of virus and vaccine hesitancy emphasise the need to remain vigilant. Given the routine practice of vital sign recording in the local context, the identification of abnormal vital signs on admission may provide a cost-effective, prompt means for prognostication of patients admitted with COVID-19, with its resultant guidance on management and referral decisions. This study aimed to evaluate the association between six routinely measured vital signs (oxygen saturation, respiratory rate, blood pressure, heart rate, glucose and temperature) and COVID-19 mortality, and secondarily to derive potential applications for South Africa and other developing countries.

## Methods

### Study design and setting

A retrospective cross-sectional study was conducted at King Edward VIII Hospital (KEH) - a tertiary institution situated in KwaZulu-Natal, South Africa. The authors acknowledge the original methods description used in a previous study titled ‘Hyperglycaemia, diabetes mellitus and COVID-19 in a tertiary hospital in KwaZulu-Natal’ from which portions of the current methodology was derived [21].

### Participants

All patients older than 13 years of age hospitalised to KEH with laboratory-confirmed SARS-CoV-2 infection during the period 1 June 2020 to 31 September 2020 were included in the study sample. Key exclusion criteria included patients without necessary information available.

### Data collection and ethics

Ethics approval for this study was obtained from the University of KwaZulu-Natal Biomedical Research and Ethics Committee (BREC/00002069/2020) and the Department of Health, together with appropriate site approval prior to commencement. Data was fully anonymised prior to collection and analysis. Informed consent was waived by the ethics committee due to the retrospective nature of the study. Research methods were conducted in accordance with relevant guidelines and regulations.

The following data was obtained from medical records of participants, and analysed as per aims of the study:

1. Basic demographics:
  Age
  Gender
  Race
2. Vital signs on admission:
  Heart rate (beats/minute)
  Systolic blood pressure (mmHg)
  Diastolic blood pressure (mmHg)
  Respiratory rate (breaths/minute)
  Oxygen saturation (%)
  Random glucose (mmol/l)
  Temperature (°C)
3. Comorbidities
4. Outcome:
  Demise/No demise
5. Duration to death/discharge (days)

### Statistical analysis

The data collected was analysed with SPSS version 27.0. The Shapiro-Wilk test was used to determine the distribution of data. Quantitative data was presented as mean (standard deviation) (SD) or median (interquartile range)(IQR) depending on the distribution of the data, and compared using Student’s t-test or Mann-Whitney U-test respectively. Categorical data was presented as frequencies and percentages and compared using chi squared tests. For univariate analysis of possible predictors of mortality, receiver operating characteristic (ROC) curve analyses for continuous variables were utilised, as well as cross-tabulation with the chi squared test for categorical variables. Factors predictive of mortality using univariate analysis were entered into multivariate analysis. The multivariate model, with vital signs identified as significant on univariate analysis, underwent ROC curve analysis to evaluate predictive ability (with and without inclusion of age). A log rank test was performed to evaluate the difference in survival rates for two different categories of oxygen saturation. A *p* value of < 0.05 was regarded as statistically significant.

### Study procedure

Participants were divided into survivors and non-survivors and comparisons made between the two cohorts in terms of admission vital signs. Parameters compared included admission heart rate, systolic blood pressure, diastolic blood pressure, respiratory rate, oxygen saturation, random glucose and temperature. Oxygen saturation was measured using pulse oximetry. The primary outcome of interest was inpatient-mortality.

### Definitions

COVID-19 was defined by a positive SARS-CoV-2 polymerase chain reaction (PCR) laboratory result. A non-survivor was defined as a participant that demised during period of hospital stay, whereas a survivor was defined as a participant that was either discharged home, down referred, or up referred without demise during period of hospital admission.

## Results

### Demographics

The total cohort comprised 236 participants – 183 (77.54%) survivors and 53 (22.46%) non-survivors. The baseline characteristics demonstrated a mean age of 53.08(16.96) years with a female (59.75%) and Black African (87.71%) preponderance. Table 1 demonstrates comparison between survivors and non-survivors in terms of demographics and comorbidity rates. The mean age of the non-survivor cohort was 64.36(13.51) years with majority of patients in the 60–79 year age groups. Conversely, the mean age of the survivor cohort was 49.81(16.48) years with most patients falling in the 40–59 year age groups. Both groups had significantly more females and Black Africans (*p* < 0.05).

**Table 1:** Demographics and comorbidities – comparison between survivors and non-survivors

	Total (*n* = 236)	Survivors (*n* = 183)	Non-Survivors (*n* = 53)	*p* value
	f (%)	f (%)	f (%)	
Age (years)				
10–19	5 (2.12)	5 (2.73)	0 (0)	0.025
20–29	21 (8.9)	20 (10.93)	1 (1.89)	< 0.001
30–39	27 (11.44)	25 (13.66)	2 (3.77)	< 0.001
40–49	43 (18.22)	37 (20.22)	6 (11.32)	< 0.001
50–59	50 (21.19)	43 (23.5)	7 (13.21)	< 0.001
60–69	49 (20.76)	32 (17.49)	17 (32.08)	0.032
70–79	29 (12.29)	14 (7.65)	15 (28.3)	0.853
80–89	9 (3.81)	6 (3.23)	3 (5.66)	0.317
90–99	3 (1.27)	1 (0.55)	2 (3.77)	0.564
Total	236	183	53	
Gender				
Female	141 (59.75)	108 (59.02)	33 (62.26)	< 0.001
Male	95 (40.25)	75 (40.98)	20 (37.74)	< 0.001
Total	236	183	53	
Race				
Black African	207 (87.71)	163 (89.07)	44 (83.02)	< 0.001
White	8 (3.39)	5 (2.73)	3 (5.66)	0.48
Coloured	1 (0.42)	1 (0.55)	0 (0)	0.317
Asian	20 (8.47)	14 (7.65)	6 (11.32)	0.074
Total	236	183	53	
Comorbidities				
Diabetes	79 (33.47)	58 (31.69)	21 (39.62)	< 0.001
Hypertension	95 (40.25)	59 (32.24)	36 (67.92)	0.018
COPD/Asthma	12 (5.08)	9 (4.92)	3 (5.66)	0.083
HIV	68 (28.81)	56 (30.6)	12 (22.64)	< 0.001

### Vital signs

**Fig. 1:**
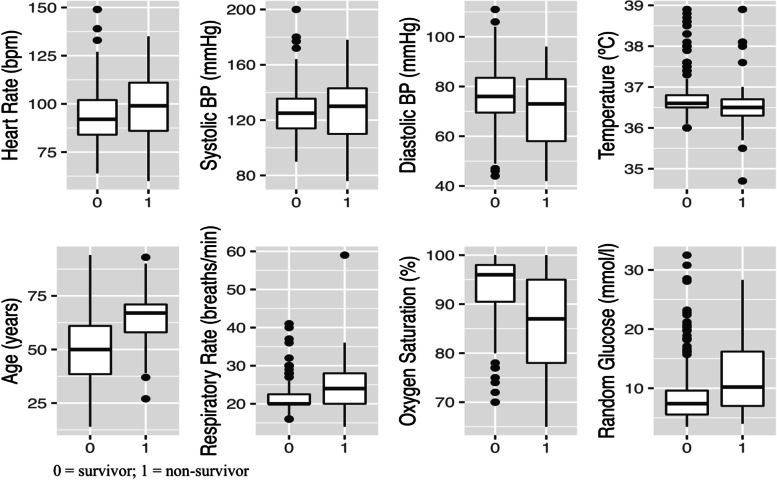
Boxplots comparing survivors and non-survivors across eight predictor variables on admission (heart rate, systolic BP, diastolic BP, temperature, age, respiratory rate, oxygen saturation, random glucose)

Table 2 demonstrates comparison between survivors and non-survivors in terms of admission vital signs. The Shapiro-Wilk test revealed a non-parametric distribution of data for oxygen saturation, respiratory rate, systolic blood pressure, heart rate, temperature and glucose; whereas a parametric distribution was observed for diastolic blood pressure. Overall, the non-survivor group demonstrated a significantly lower median/mean for admission oxygen saturation and diastolic blood pressure, and a significantly higher median for admission respiratory rate and glucose. No significant difference in admission heart rate, systolic blood pressure and temperature was observed between survivors and non-survivors (Fig. 1).

**Table 2:** Admission vital signs - comparison between survivors and non-survivors

	Total (*n* = 236)		Survivors (*n* = 183)		Non-Survivors (*n* = 53)		*p* value
	Median (IQR)	Mean (SD)	Median (IQR)	Mean (SD)	Median (IQR)	Mean (SD)	
Oxygen Saturation (%)	95 (88–98)		96 (90–98)		87 (78–95)		< 0.001
Respiratory rate (breaths/minute)	21 (20–24)		20 (20–23)		24 (20–28)		< 0.001
Heart rate (bpm)	94 (85–103.75)		92 (84–102)		99 (85.5–111)		0.067
Systolic blood pressure (mmHg)	126 (113–137)		125 (114–136)		130 (107.5–143)		0.494
Diastolic blood pressure (mmHg)		75.06 (12.87)		76.3 (12.07)		70.79 (14.66)	0.006
Temperature (°C)	36.6 (36.4–36.8)		36.6 (36.5–36.8)		36.5 (36.25–36.7)		0.140
Glucose (mmol/l)	7.55 (5.7–12.05)		7.4 (5.5–9.8)		10.2 (6.95–16.25)		< 0.001

### Univariate analysis

#### Continuous variables

Table 3 demonstrates univariate association between continuous predictor variables and in-hospital mortality. The level of effect was such that in-hospital mortality was favoured by a reduced oxygen saturation and diastolic blood pressure, and an elevated respiratory rate, glucose and age.

**Table 3:** Univariate receiver operator curve (ROC) analysis of continuous variables associated with in-hospital mortality

Continuous variable	Area under the curve (AUC)	95% CI	*p* value
Oxygen saturation (%)	0.737	0.655–0.820	< 0.001
Respiratory rate (breaths/minute)	0.658	0.569–0.747	< 0.001
Heart rate (bpm)	0.582	0.491–0.674	0.068
Systolic blood pressure (mmHg)	0.531	0.431–0.630	0.495
Diastolic blood pressure (mmHg)	0.596	0.502–0.691	0.033
Temperature (°C)	0.566	0.473–0.659	0.143
Glucose (mmol/l)	0.662	0.580–0.745	< 0.001
Age (years)	0.759	0.688–0.830	< 0.001

#### Categorical variables

Table 4 demonstrates univariate association between categorical variables and in-hospital mortality. Only hypertension was found to be significantly associated with mortality (unadjusted Odds Ratio(OR) 4.451; 95% Confidence Interval(CI) 2.312–8.566; *p* < 0.001).

**Table 4:** Univariate cross-tabulation analysis of categorical variables associated with in-hospital mortality

Categorical variable	Unadjusted OR	95% CI	x^2^ test	*p* value
Gender	1.146	0.611–2.149	0.180	0.671
Diabetes	1.414	0.751–2.662	1.160	0.281
Hypertension	4.451	2.312–8.566	21.759	< 0.001
COPD/Asthma	1.160	0.303–4.448	0.047	0.828
HIV	0.664	0.324–1.358	1.269	0.260

### Multivariate analysis

**Fig. 2:**
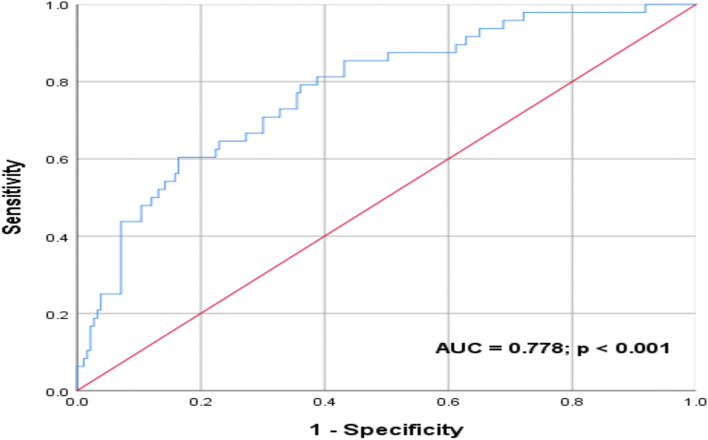
ROC curve – predictive value of oxygen saturation, respiratory rate, diastolic blood pressure and glucose on COVID-19 in-hospital mortality

**Fig. 3:**
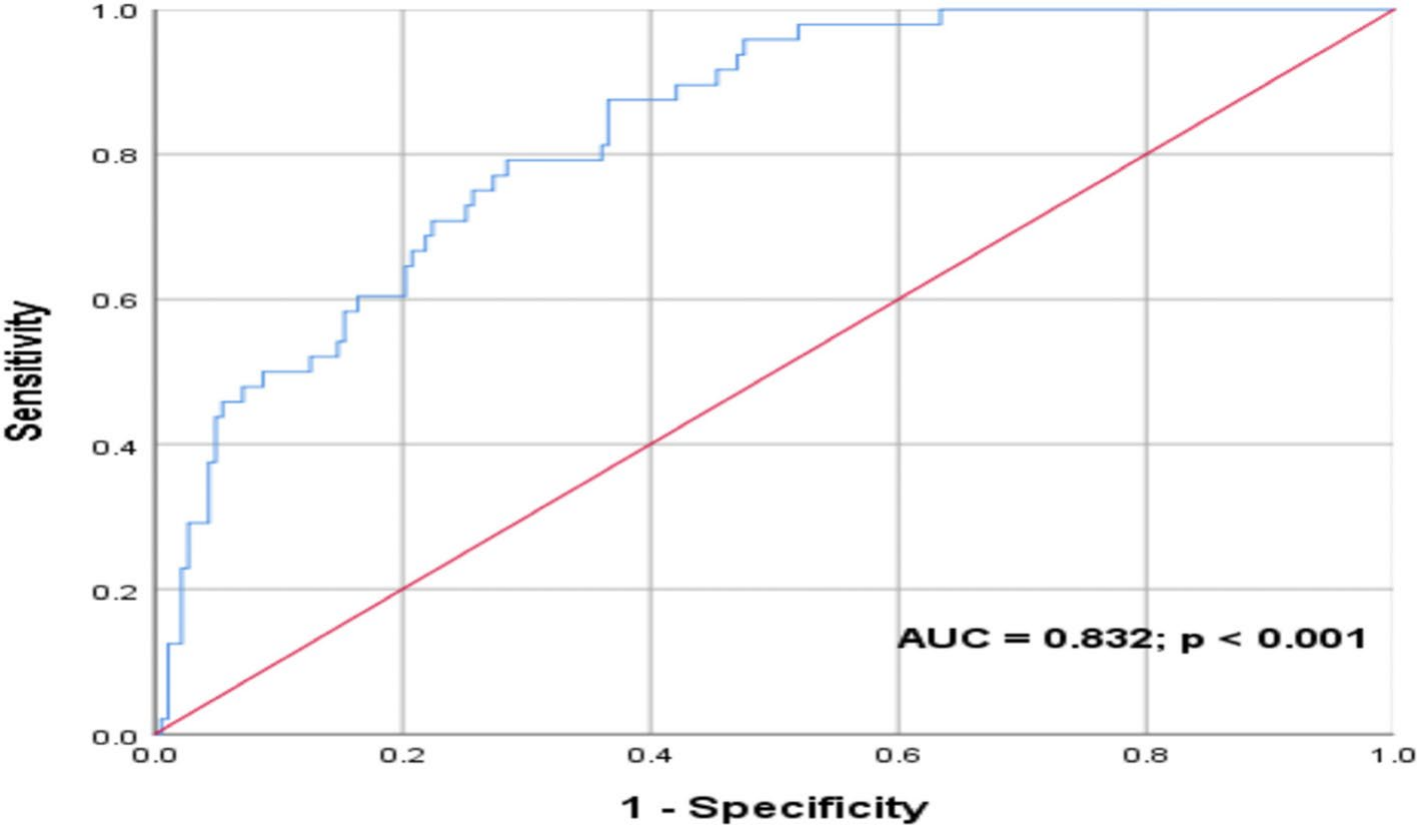
ROC curve – predictive value of age, oxygen saturation, respiratory rate, diastolic blood pressure and glucose on COVID-19 in-hospital mortality

All variables identified through univariate analysis as significantly correlating with mortality were then entered into multivariate analysis (Table 5). Lower oxygen saturation and higher age were significantly associated with mortality on multivariate analysis – for every 1 % increase in oxygen saturation on admission, the odds of mortality decreased by 7.8%; and for every one-year increase in age, the odds of mortality increased by 4.7%. Age was excluded from this multivariate regression model and a ROC curve analysis was conducted on the remaining predictor variables in Table 5 to assess the predictive accuracy of these vital signs (oxygen saturation, respiratory rate, diastolic blood pressure and glucose). An area under the curve (AUC) of 0.778 was revealed with a significant *p* value of < 0.001 (Fig. 2). Age was then included in this analysis to assess its influence, resulting in an improvement in the predictive accuracy of the model (AUC 0.832; *p* < 0.001) (Fig. 3).

**Table 5:** Multivariate logistic regression analysis of identified univariate predictors of mortality

Variable	Level of effect	OR	95% CI	*p* value
Oxygen saturation (%)	Lower oxygen saturation favouring mortality	0.922	0.880–0.965	0.001
Respiratory rate (breaths/minute)	Higher respiratory rate favouring mortality	1.019	0.935–1.111	0.662
Diastolic blood pressure (mmHg)	Lower diastolic blood pressure favouring mortality	0.979	0.951–1.008	0.160
Glucose (mmol/l)	Higher glucose favouring mortality	1.042	0.976–1.113	0.220
Age (years)	Higher age favouring mortality	1.047	1.016–1.080	0.003

**Fig. 4:**
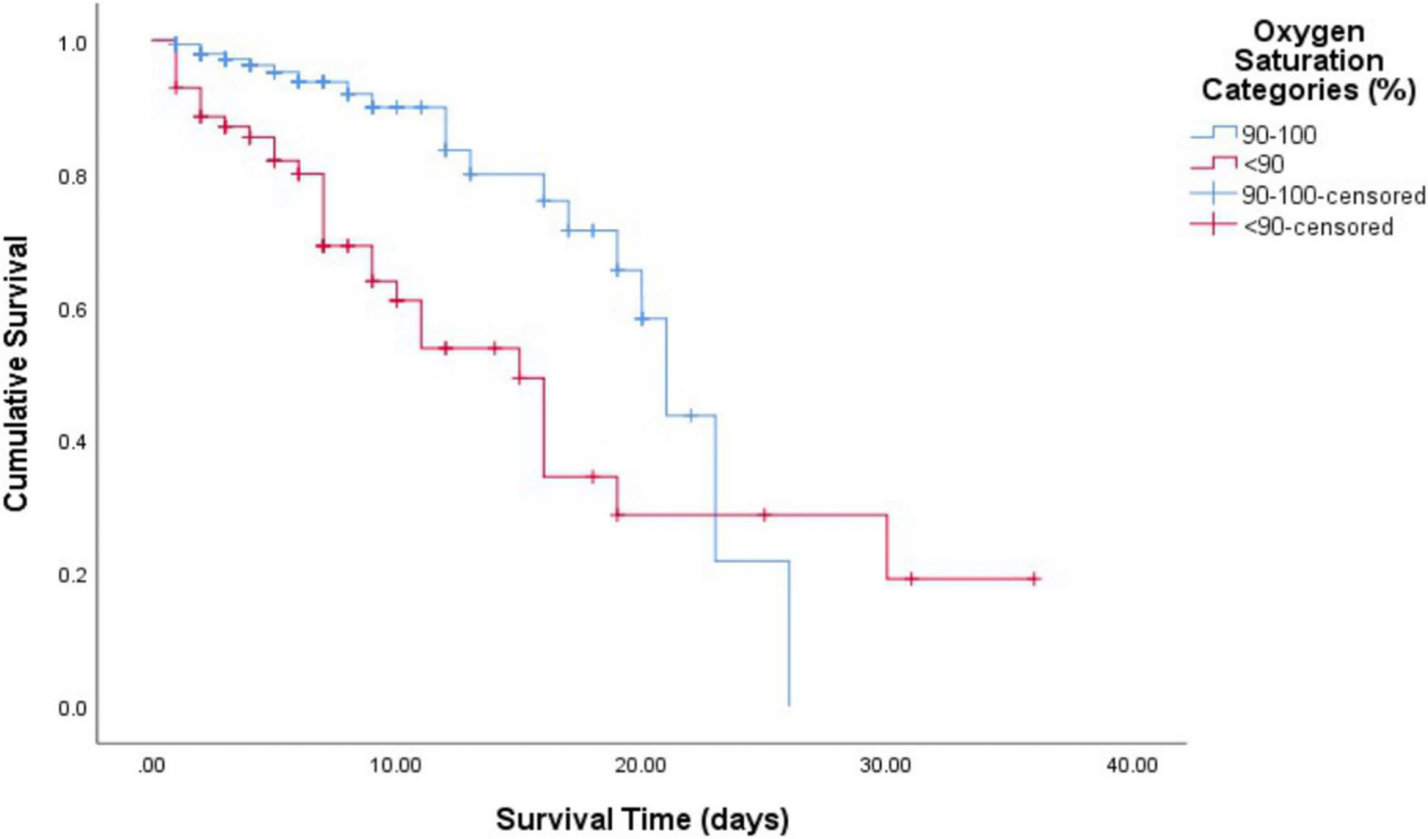
Kaplan Meier Survival Analysis – Time to death (days) for two categories of admission oxygen saturation (%)

Figure 4 demonstrates difference in survival rates amongst those with an admission oxygen saturation < 90% vs. ≥90%. A log rank test revealed significantly lower survival rates in patients with a baseline oxygen saturation < 90% (*p* = 0.001), with a median time to death of 15 days (95% CI 11.266–18.734). For those with an initial saturation ≥ 90%, the median time to death was 21 days (95% CI 18.986–22.506).

Overall for non-survivors, the mean (SD) oxygen saturation values measured by pulse oximetry (Sp02) were greater than those measured by arterial blood gas (ABG) analysis (Sa02), with the greatest difference observed for oxygen saturation group < 80% (Table 6). Using a cut-off of < 90%, pulse oximetry had a sensitivity of 96.7% and specificity of 100%.

**Table 6:** Oxygen saturation amongst non-survivors – comparison between pulse oximetry and arterial blood gas analysis

Group	Method	Mean (SD)
Total non-survivors	Pulse oximetry	86 (9.94)
	ABG	84.79 (10.9)
Oxygen saturation ≥ 90%	Pulse oximetry	95.68 (3.18)
	ABG	95.67 (3.01)
80% ≤ Oxygen saturation < 90%	Pulse oximetry	85.87 (1.92)
	ABG	85.33 (2.09)
Oxygen saturation < 80%	Pulse oximetry	73 (4.45)
	ABG	70.64 (5.54)

## Discussion

This study demonstrates the value of admission vital signs in predicting mortality in patients hospitalised with COVID-19. Lower oxygen saturation, elevated respiratory rate, lower diastolic blood pressure and elevated glucose were found to be significantly associated with in-hospital mortality and comprised components of a promising predictive model. Numerous studies describe the relationship between oxygen saturation, respiratory rate and glucose on outcome [3–9]; however, the significance of admission diastolic blood pressure is a relatively novel finding. Although this study aimed to assess the influence of admission *vital signs* as a predictor of COVID-19 outcome, age is another variable that is easily available on initial consultation and was included into the prediction model, yielding an improvement in predictive ability.

COVID-19 remains a major healthcare concern globally and in South Africa. Vital signs are routinely measured for all patients presenting to healthcare facilities across all levels of care, and may serve as an early marker of poor prognosis. Global studies reveal important associations with severity and outcome, which are, however, not necessarily generalisable to the local context given the heterogeneity of the South African population. This study aimed to address the paucity of such data, and secondarily to derive important practical applications for other developing countries as well.

The vital signs evaluated in this study are routinely measured on initial consultation and may provide an early indication of patients with COVID-19 requiring more intensive in-hospital monitoring and treatment. Moreover, the heavy burden of this disease in the public healthcare sector has resulted in patients being managed across all levels of care – district, regional and tertiary – despite substantial difference in resources and skills. Thus, the early identification of patients with vital signs predictive of a poor prognosis may allow for prompt referral to an appropriate center.

Lower oxygen saturation on admission was found to be an independent risk factor for mortality. For every 1 % increase in admission oxygen saturation, the odds of mortality decreased by 7.8%. Given the phenomenon of silent hypoxaemia described in patients with COVID-19, this may represent delayed presentation to a healthcare facility [22]. Although this study focused on hospitalised patients, home oxygen saturation monitoring in non-hospitalised patients with mild infection may also serve as a valuable tool by providing an early warning sign to patients. A large retrospective South African study by Nematswerani et al. yielded significantly lower mortality rates in patents who utilised a pulse oximeter to monitor oxygen saturation at home vs. those that did not [22]. Given the potential benefits, consideration ought to be given to provision of pulse oximeters to high-risk patients with COVID-19 being managed at home, with a low threshold for presentation to hospital.

Measurement of blood oxygen saturation using pulse oximetry is a useful non-invasive tool, however reports of overestimation of true arterial oxygen saturation in darkly pigmented patients raise some concern over its utility in African populations [23]. Even though the non-survivors in this study demonstrated an overall higher mean oxygen saturation when measured by pulse oximetry versus arterial blood gas analysis (86% vs 84.79% respectively), the difference was small, especially at higher oxygen saturations, suggesting that pulse oximeters may be of value in home monitoring of stable patients even in the local population. Further research in this regard is necessary, in predominantly African populations, to identify the ideal method of oxygen saturation measurement for risk stratification - especially at lower oxygen saturation levels.

Several studies concur with our findings - revealing a significant relationship between a low oxygen saturation, elevated glucose and elevated respiratory rate on admission and adverse COVID-19 outcomes [3–9]; however, the significance of diastolic blood pressure is a novel finding. A study by Fei-Ka Li et al. revealed no significant difference in the mean blood pressure (systolic and diastolic) between patients with critical vs. severe disease with COVID-19. However greater systolic and diastolic blood pressure *variation* was observed in those with critical disease, and both systolic and diastolic blood pressure variation indices showed positive association with worse outcomes (*p* = 0.02–0.03 and *p* = 0.06–0.08 respectively) [24]. However, despite revealing a significant association with mortality on univariate analysis in our study, diastolic blood pressure lost its statistical significance on multivariate analysis – possibly suggesting confounding.

This study failed to demonstrate any significant association between systolic blood pressure, heart rate and temperature on admission *and* in-hospital mortality. In keeping with this, most other studies found no significant association between admission fever and mortality [11, 12]. Tharakan et al., however, analysed body temperature in 7614 patients with COVID-19 and identified hypothermia as in important marker of poor prognosis in patients with an admission temperature < 36 °C, and even more so < 35.5 °C [12].

Hypotension and tachycardia are key features of advanced disease and have been shown to indicate a poor prognosis in various generic sepsis-related risk scores [14]. However, a study by Caillon et al. demonstrated high systolic blood pressure measurement on admission as an important component of mortality predication models [17]. Advanced age (with comorbid hypertension) is a major risk factor for mortality in COVID-19 patients, thus it is uncertain whether this represents the burden of uncontrolled hypertension in the deceased population or occurred as a consequence of systemic inflammation and/or interference with angiotensin-converting enzyme 2 (ACE-2) enzymatic activity by SARS-CoV-2 [17].

Interestingly, although cardiac involvement usually occurs in association with systemic disease, there exists reports of isolated pericardial involvement with SARS-CoV-2 infection – demonstrating the expanding spectrum of cardiac affectation in COVID-19 [25].

The utilisation of vital signs in disease and outcome prediction has proven to be of immense value. With regards to sepsis prediction in critically ill adults, Mohammed et al. developed a prediction model utilising minute-to-minute physiological data only (heart rate, blood pressure and respiratory rate) and was able to accurately predict sepsis a mean of 17.4 h before sepsis onset with an average test accuracy of 83% [26]. Van Wyk et al. similarly developed a prediction model comprising a minimal set of continuous routinely measured vital signs only – heart rate, respiratory rate, systolic and diastolic blood pressure, temperature and oxygen saturation – and was able to predict sepsis a mean of 5 h prior to onset [27]. Furthermore, the addition of white cell count did not improve model sensitivity. This emphasises that effective prediction models comprising vital signs only exist, and may serve as a critical predictive modelling tool in the realm of COVID-19 as well.

Numerous COVID-19 risk prediction models have been developed; however, they are not necessarily applicable to the context of a developing country given the resource constraints. Further studies with larger cohorts need to be conducted to assess the performance of a prediction model comprising admission vital signs, together with appropriate external validation. Ideally, the models should be sensitive to the resource limitations of the public healthcare sector – comprising variables that are consistently measured and readily available, allowing for utilisation across various levels of care. Despite nearly 2 years since inception, COVID-19 remains a prime healthcare concern globally and in South Africa. Resource limitations coupled with the emergence of novel strains of virus and vaccine hesitancy emphasise the need to remain vigilant. The development and implementation of COVID-19 risk prediction models, comprising easily available parameters, may serve as a vital tool in upcoming waves of infection in resource-constrained settings. Further research in this regard would be forthcoming.

### Study limitations

Our study comprised a relatively small sample size spanning June–September 2020. Ever since, newer strains of virus have emerged with postulated differences in behaviour. Thus, the study may not represent characteristic of the current and/or future strains of SARS-CoV-2. Again, highlighting the need for continued research of such nature. Furthermore, the study was limited to the public healthcare sector and did not equally represent race, age groups and socioeconomic status of the population. In addition to admission vital signs, other parameters (comorbidities etc.) are also readily available on initial consultation and may have provided value when integrated into the risk prediction model. The aim of this study is to demonstrate the importance of admission vitals in early risk stratification and its association with COVID-19-related mortality. More extensive machine learning algorithms are beyond the scope of objectives. Hopefully this article prompts further research and development of machine learning models (using larger sample sizes) specifically comprising easily available parameters and targeting developing populations with resource limitations.

## Conclusion

COVID-19 remains a major healthcare concern globally, and more so in developing countries with numerous resource and logistical limitations. A multivariate regression model comprising readily available information - admission oxygen saturation, respiratory rate, glucose and diastolic BP (with/without age) - demonstrated promising predictive capacity, and may provide a cost-effective means for early prognostication of patients admitted with COVID-19 in resource-limited settings. Further studies with larger cohorts need to be conducted to assess the strength of this association and its generalisability.

## Supplementary Information

**Additional file 1.**

## Data Availability

All data generated or analysed during this study are included in this published article and its supplementary information files.

## Abbreviations

COVID-19: Coronavirus Disease 2019
SARS-CoV-2: Severe acute respiratory syndrome coronavirus 2
CI: Confidence interval
OR: Odds ratio
SOFA: Sequential Organ Failure Assessment
qSOFA: Sequential Organ Failure Assessment (quick)
SIRS: Systemic Inflammatory Response Syndrome
MEWS: Modified Early Warning Score
NEWS: National Early Warning Score
KEH: King Edward VIII Hospital
SD: Standard deviation
IQR: Interquartile range
ROC: Receiver operating characteristic
AUC: Area under the curve
PCR: Polymerase chain reaction
COPD: Chronic obstructive pulmonary disease
HIV: Human Immunodeficiency Virus
BP: Blood pressure
ACE-2: Angiotensin-converting enzyme 2
ABG: Arterial blood gas
